# Comparison of the Transcriptomes of Long-Term Label Retaining-Cells and Control Cells Microdissected from Mammary Epithelium: An Initial Study to Characterize Potential Stem/Progenitor Cells

**DOI:** 10.3389/fonc.2013.00021

**Published:** 2013-02-15

**Authors:** Ratan K. Choudhary, Robert W. Li, Christina M. Evock-Clover, Anthony V. Capuco

**Affiliations:** ^1^Department of Animal and Avian Sciences, University of MarylandCollege Park, MD, USA; ^2^Bovine Functional Genomics Laboratory, USDA-ARSBeltsville, MD, USA

**Keywords:** label retention, mammary stem cells, mammary progenitor cells, stem cell markers, laser microdissection

## Abstract

**Background:** Previous molecular characterizations of mammary stem cells (MaSC) have utilized fluorescence-activated cell sorting or *in vitro* cultivation of cells from enzymatically dissociated tissue to enrich for MaSC. These approaches result in the loss of all histological information pertaining to the *in vivo* locale of MaSC and progenitor cells. Instead, we used laser microdissection to excise putative progenitor cells and control cells from their *in situ* locations in cryosections and characterized the molecular properties of these cells. MaSC/progenitor cells were identified based on their ability to retain bromodeoxyuridine for an extended period.

**Results:** We isolated four categories of cells from mammary epithelium of female calves: bromodeoxyuridine label retaining epithelial cells (LREC) from basal (LRECb) and embedded layers (LRECe), and epithelial control cells from basal and embedded layers. Enriched expression of genes in LRECb was associated with stem cell attributes and identified WNT, TGF-β, and MAPK pathways of self renewal and proliferation. Genes expressed in LRECe revealed retention of some stem-like properties along with up-regulation of differentiation factors.

**Conclusion:** Our data suggest that LREC in the basal epithelial layer are enriched for MaSC, as these cells showed increased expression of genes that reflect stem cell attributes; whereas LREC in suprabasal epithelial layers are enriched for more committed progenitor cells, expressing some genes that are associated with stem cell attributes along with those indicative of cell differentiation. Our results support the use of DNA label retention to identify MaSC and also provide a molecular profile and novel candidate markers for these cells. Insights into the biology of stem cells will be gained by confirmation and characterization of candidate MaSC markers identified in this study.

## Introduction

In female mammals, growth and development of mammary glands occur primarily postnatally, with mammary function in the mature animal being tightly coupled to reproductive strategy. This dictates cycles of mammary growth, differentiation, lactation, and regression, during which mammary stem cells (MaSC) provide for the lineages of luminal and basal (myoepithelial) epithelial cells in the ducts and alveoli. Although mice have provided the primary model for study of mammary growth and development, a single model species cannot provide comprehensive knowledge. Because mammary glands of prepubertal calves have a tissue architecture resembling that of the prepubertal human breast more closely than does mouse (Capuco et al., 2002), cows provide an additional experimental model for human breast development. Increased knowledge of MaSC is directly applicable to agriculture and the development of management schemes to enhance the lifetime productivity of dairy cows and other species.

A method that has been used to identify MaSC is based upon the capacity of these cells to retain 5-bromo-2′-deoxyuridine (BrdU) labeled DNA for an extended period (Kenney et al., 2001; Welm et al., 2002; Smith, 2005; Capuco, 2007). Retention of labeled DNA strands may be attributed to the ability of stem cells to retain the parental DNA strand during asymmetric cell division (Cairns, 1975) or to quiescence of the stem cell population such that the DNA label is not diluted by frequent cell divisions (Klein and Simons, 2011). During rapid mammary growth in the mouse, label retaining epithelial cells (LREC) appear to retain label by asymmetric distribution of DNA strands, as evidenced by a rapid proliferation index of the LREC (Smith, 2005). During periods of low mammary proliferation, quiescence of the stem cell population may account for retention of label. LREC are enriched in populations that exhibit MaSC capacity, i.e., the ability to regenerate mammary epithelium upon transplantation into the cleared mammary fat pad of syngeneic mice (Welm et al., 2002).

We previously reported that LREC in mammary epithelium of calves were localized in the basal layer (LRECb) and in the embedded (LRECe) layers between the basal and luminal cells of a multilayered epithelium (Capuco, 2007; Capuco et al., 2009). The LREC in bovine mammary gland appeared to have a modest proliferation rate in which 5.4% of LREC co-expressed Ki-67 (Capuco, 2007). LRECb were estrogen receptor-α (ESR1) -negative and hypothesized to be MaSC, whereas the LRECe were a mixed population of ESR1-positive and -negative cells that were hypothesized to be progenitor cells (Capuco, 2007; Capuco et al., 2009). The estrogen receptor status of MaSC is of considerable interest because of the importance of estrogens for MaSC function, mammary ductal growth, and tumorigenesis. MaSC of mouse and human are ESR1-negative (Anderson and Clarke, 2004; Asselin-Labat et al., 2006; Sleeman et al., 2007; Lamarca and Rosen, 2008).

Morphological evidence suggests that MaSC are basally localized within the mammary epithelium, typically underlain by cytoplasmic extensions of epithelial cells and in close proximity to ESR1-positive epithelial cells (Smith and Chepko, 2001; Brisken and Duss, 2007). However, MaSC have not been fully characterized due to technical limitations inherent in stem cell identification and in isolation of cells from known locations within the mammary epithelium. Based on fluorescence-activated cell sorting with multiple biomarkers and use of mammary transplantation methods to evaluate multi-lineage potency, Shackleton, Stingl, and colleagues obtained and characterized a population of cells, from enzymatically dispersed mammary tissue, that was enriched for MaSC (Shackleton et al., 2006; Stingl et al., 2006). Critical to the success of this pioneering approach was use of markers to deplete the population of hematopoietic (CD45 and TER119) and endothelial cells (CD31), as well as markers to select epithelial cells (CD29, CD49f), likely from a basal location, that expressed heat stable antigen (CD24). Another approach utilized for enrichment and characterization of human MaSC involved characterization of mammary epithelial cells that possess multipotency potential *in vitro* (Dontu et al., 2003).

Cell sorting techniques have also been applied to suspensions of bovine mammary cells in an attempt to enrich for MaSC. Motyl et al. (2011) isolated and evaluated gene expression in a population of mammary cells that were isolated on the basis of SCA1 expression and showed up-regulation of genes that are characteristic of hematopoietic cells. However, because accompanying micrographs clearly show that most SCA1-positive cells were in the mammary stroma and methods to enrich for mammary epithelial cells were not employed, the gene expression profile likely cannot be attributed to MaSC. Furthermore, previous research indicates the likelihood of hematopoietic cells populating the mammary stem cell niche is highly unlikely (Niku et al., 2004). Research by Martignani et al. (2010) utilized aldehyde dehydrogenase (ALDH) activity as a selection criterion for cell sorting and demonstrated that cells with low ALDH activity were capable of regenerating functional structures of mammary epithelium within collagen gels implanted beneath the kidney capsule of immunodeficient mice. This latter study not only provides data pertaining to characteristics of bovine bipotent progenitor cells, but validates a means to assess such potency. Most recently, Rauner and Barash (2012) used the multiparameter cell sorting technique developed for enrichment of murine MaSC (Shackleton et al., 2006) to obtain and characterize four populations of mammary epithelial cells from dissociated bovine mammary gland. The differentiation and growth potential of the cells were assessed by *in vitro* colony formation and mammosphere assays. This study confirmed many of the general aspects of MaSC/progenitor cells evident in mouse and human studies. The four populations included putative bovine MaSC (CD24^med^CD49f^pos^) that were bipotent (myoepithelial and luminal) and possessed a high growth rate; basal bipotent progenitors with medium growth rate and low sphere generating potential; luminal unipotent progenitors with low growth rate; and luminal unipotent cells with very limited proliferative activity. Although putative MaSC typically possessed little or no ALDH activity, as reported previously (Martignani et al., 2010), 0.4% of total viable cells expressed high ALDH activity, which they hypothesized represent the MaSC population.

In addition to issues pertaining to the isolation of MaSC from a mixed suspension of mammary cells, all previous studies have evaluated MaSC after removing them from their stem cell niche, i.e., the microenvironment of surrounding signaling molecules and other non-cellular components that support stem cell function and survival. We have taken an approach that retains histological information by characterizing gene expression in putative MaSC directly after their *in situ* excision from the mammary epithelium. The histological location of all cells interrogated was known.

In the present study, putative stem and progenitor cells (LREC) were identified and excised from cryosections using laser microdissection. It must be recognized that identification of putative MaSC and progenitor cells on the basis of long-term retention of DNA label is to select the cells based upon their life-history (i.e., the extent of label retention represents an integration of the cell’s past proliferation and differentiation events). Consequently, one would anticipate that selecting putative MaSC and progenitor cells based on label retention is likely to represent enrichment for these cell populations. In this study, LREC and neighboring epithelial control (non-LREC) cells were excised from two different locations: basal and embedded layers of the mammary epithelium. We hypothesized that LRECb are enriched for MaSC whereas LRECe are enriched for more committed progenitor cells, and that by comparing the transcriptomes of these cells with neighboring control cells we would obtain molecular profiles and biomarkers for MaSC and progenitor cells. Results are consistent with these hypotheses and provide novel candidate markers for MaSC and progenitor cells.

## Materials and Methods

### Experimental animals and mammary tissue

Use of animals for this study was approved by the Beltsville Agricultural Research Center’s Animal Care and Use Committee. Tissues for this study were obtained from five Holstein heifers at approximately 5 months of age (4.8 ± 0.05, mean ± SE). At approximately 3 months of age, heifers were injected intravenously with BrdU (Sigma-Aldrich Co., St. Louis, MO, USA) for five consecutive days. BrdU was administered in a saline solution containing 20 mg BrdU/ml (0.9% sodium chloride; pH 8.2) at a dosage of 5 mg/kg body weight, as described previously (Capuco, 2007). Heifers were sacrificed humanely at the Beltsville Agricultural Research Center abattoir 45 days after the last BrdU injection. Mammary tissue (∼5 mm × 5 mm × 5 mm) was collected from the outer parenchymal region (region in close proximity to the border with mammary fat pad) of a rear mammary gland. Individual samples were immediately embedded in OCT compound (Sakura, Torrance, CA, USA), frozen in liquid nitrogen vapor and stored at −80°C until use.

Cryosections of 8 μm thickness were thaw-mounted on ultraviolet-irradiated PEN slides (Leica AS, Wetzlar, Germany) and stored at −80°C until BrdU immunostaining and laser microdissection within 8 days. Mammary tissues harvested for histological validation of microarray data were fixed overnight in 10% neutral buffered formalin at 4°C and then stored in 70% ethanol until further processing. Tissues were then dehydrated and embedded in paraffin according to standard techniques and sectioned at 5 μm thickness onto Superfrost-plus™ slides (Erie Scientific Co., Portsmouth, NH, USA).

### BrdU immunostaining to identify putative MaSC

**Figure 1:**
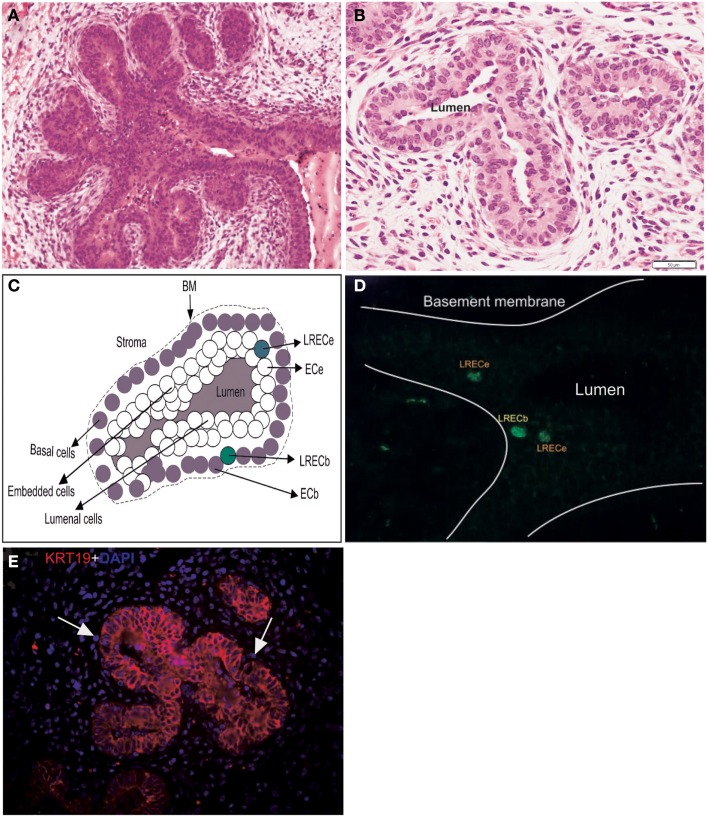
**Characteristics and dissection of the mammary epithelium of a prepubertal heifer**. **(A,B)** Micrographs of the terminal ductular units of a prepubertal bovine mammary gland illustrating the arborescent nature of these terminal ducts and their multilayered epithelium. **(C)** Diagrammatic representation of the mammary epithelium illustrating the four classes of cells dissected. BM, basement membrane. **(D)** Micrograph of cryosections stained for BrdU antigen prior to laser microdissection, localization of LRECb and LRECe are depicted. **(E)** Micrograph of epithelium of terminal ductular unit stained for KRT19. The basal layer consists of cells that are predominantly KRT19-negative.

Putative MaSC were identified as those cells in cryosections that retained BrdU label (Figure 1D), visualized using an optimized method for BrdU immunostaining that retains RNA quality in tissue cryosections (Choudhary et al., 2010). Sections were individually processed immediately before laser microdissection. The cryosections were fixed in acetone/polyethylene glycol 300 (9:1 v/v) at −20°C for 2 min and air dried for 1 min and then incubated with 0.5% methyl green for 2 min at room temperature (RT). After a brief wash (10 s) with nuclease-free phosphate buffered saline (nfPBS), 400 μl of a pre-warmed solution of 70% deionized formamide in nfPBS was pipetted onto the tissue and the section incubated at 60°C for 4 min. The section was washed with antibody dilution buffer (nfPBS with 1% normal goat serum and 0.1% triton-X 100) at 4°C on a metal plate kept on ice to prevent re-annealing of DNA strands and then incubated with mouse monoclonal anti-BrdU antibody conjugated to Alexa 488 (Clone PRB-1, 1:10 dilution, Molecular Probes, Carlsbad, CA, USA) for 5 min at RT in the dark. The section was washed briefly before counterstaining with propidium iodide (2.5 μg/μl in nfPBS). Finally, the slide was washed with nuclease-free water (10 s), dehydrated in ascending concentrations of ethanol and air dried before laser microdissection.

### Laser microdissection and cDNA amplification

Immediately after staining, sections were examined and cells excised with a laser microdissection system equipped for epifluorescence microscopy (Leica AS-LMD, Mannheim, Germany). The laser setting was determined empirically and dissection performed using the 40× objective. We dissected 6–13 cells per category per heifer. For each animal, cells in a given category were collected into the cap of a 0.2 ml thin-walled PCR tube (Biozyme Scientific GmbH, Hess Oldendorf, Germany). Total processing time for immunostaining and microdissection was less than 1 h, and only one slide was processed at a time. Four categories of cells were dissected: LREC from basal (LRECb) and embedded layers (LRECe), and epithelial control cells from basal (ECb) and embedded layers (ECe). Cells within the cap were dissolved in 2 μl of lysis buffer (WT-Ovation™ One-Direct RNA Amplification System; NuGEN Technologies, Inc., San Carlos, CA, USA). The tube was capped and centrifuged for 1 min at 14,000 × *g*, after which the tube and contents were vortexed gently for 30 s and centrifuged briefly before placing on ice. First stand cDNA synthesis and amplification reaction were carried out using Ribo-SPIA-based methodology according to the manufacturer’s recommendations. Concentrations of amplified cDNA were determined spectrophotometrically (ND-1000, NanoDrop Technologies, Rockland, DE, USA). A known amount of high quality RNA (250 pg) was used as positive control for cDNA amplification. Nuclease-free water was used as a no-template control for cDNA amplification. The amplified cDNA was evaluated using RNA Nano-chips to estimate the median fragment size (Agilent Technologies, Palo Alto, CA, USA). Median fragment size for amplified samples was similar to the positive control and fell within the expected range of 100–300 bp, whereas products for the no-template control were <50 bp.

### Microarray analysis

Oligonucleotide microarray analysis was performed using a custom bovine microarray (Nimblegen, Inc., Madison, WI, USA) as described previously (Li et al., 2006). The bovine microarray consisted of 86,191 unique 60-mer oligonucleotides, representing 45,383 bovine sequences. The array design was based upon a TIGR assembly (release 11.0 from 2004). However, all 60-mer oligonucleotides on the array were annotated against current bovine RefSeq databases as well as the latest version of ENSEMBL bovine gene build v65.0 (released on December 2011^1^). After hybridization, scanning, and image acquisition, the data were extracted from the raw images using NimbleScan software (NimbleGen). A total of 21 microarrays (five animals × four categories of cells, and no-template amplification control) were used. Relative signal intensities (log2) for each feature were generated using the robust multi-array average algorithm (Irizarry et al., 2003) and data were processed based on the quantile normalization method (Bolstad et al., 2003). Only oligos that provided hybridization signal intensities for samples that exceeded 3× the signal intensity obtained with the no-template amplification control (water blank) were included in the analysis. Furthermore, only sample signal intensities exceeding twice the array background intensity (mean of lowest 3% of oligo intensities) were considered for analysis.

*P* values were calculated using a modified *t*-test. Fold changes were calculated as the ratio of the means of background-adjusted, normalized fluorescent intensity of cells of interest to their respective controls. Group-wise comparisons were performed in accordance with recommendations of the Microarray Quality Control project (Shi et al., 2006, 2008) based on *t*-test (*P* < 0.05) followed by fold change (twofold as a cutoff) to determine significance. These criteria were shown to achieve a balance of reproducibility, sensitivity, and specificity, using single or multiple microarray platforms (Shi et al., 2006, 2008; Chin et al., 2009, 2010; Wang et al., 2011). Based upon these criteria, genes that were differentially expressed were then subjected to pathway analysis (IPA, Ingenuity Systems^2^).

The microarray data discussed in this publication have been deposited in NCBI’s Gene Expression Omnibus (Edgar et al., 2002) and are accessible through GEO Series accession number GSE31541^3^.

### Realtime quantitative RT-PCR

Realtime quantitative RT-PCR (qRT-PCR) was performed using aliquots of amplified cDNA from all animals and an IQ SYBR Green Supermix kit (Bio-Rad Laboratories, Hercules, CA, USA). Each reaction was performed in a 25 μl reaction volume containing 200 nM of each amplification primer and 2 ng of cDNA. The amplification was performed in a Bio-Rad iCycler using the following protocol: 95°C – 60 s; 45 cycles of 94°C – 15 s, 61°C – 30 s, and 72°C – 30 s. A melting curve analysis was performed for each primer pair. Standards were prepared from PCR amplicons purified using the QIAquick purification kit (Qiagen Inc., Valencia, CA, USA). Product concentrations were determined using the Agilent 2100 BioAnalyzer and DNA 500 kits (Agilent Technologies) and diluted to contain 1 × 10^2^ to 1 × 10^8^ molecules/μl. Quantity of cDNA in unknown samples was calculated from the appropriate external standard curve run simultaneously with samples.

### Immunohistochemistry

Paraffin sections were dewaxed in xylene and hydrated in a graded series of ethanol to phosphate buffered saline (PBS, pH 7.4). Tissue sections were quenched with 3% H_2_O_2_ in PBS for 10 min and then washed in PBS. Antigen retrieval was performed by incubation with 70% formamide in PBS at 60°C for 5 min, or microwave heating in 10 mM Tris containing 1 mM EDTA, pH 9.0 (5 min heat, 5 min rest, 5 min heat, 25 min cooling). Sections were blocked with casein (CAS-block™, Invitrogen, Carlsbad, CA, USA). Primary antibodies NR5A2, NUP153, and HNF4A (Abcam Inc., Cambridge, MA, USA) were used at 1:200 dilution and FNDC3B (Santa Cruz, Santa Cruz, CA, USA) at 1:50. Sections were incubated with primary antibody for 2 h at RT or overnight at 4°C. After washing in PBS, sections were incubated with horseradish peroxidase-conjugated broad spectrum secondary antibody (ImmPRESS anti-mouse/anti-rabbit, Vector Labs, Burlingame, CA, USA). Positively labeled cells were visualized brown or purple using 3,3′-diaminobenzidine or ImmPACT VIP (Vector Labs), respectively. Slides were washed and then counterstained with hematoxylin or methyl green.

To determine if cells expressing FNDC3B were LREC, dual antigen labeling was performed. Tissue sections were processed as described earlier and incubated with mouse monoclonal BrdU antibody (Clone BMC 9318, 2 μg/ml; Roche Diagnostics Corp., Indianapolis, IN, USA) for 2 h at RT. Sections were then incubated with vector ImmPRESS anti-mouse polymer detection reagent (Vector Labs) for 20 min, followed by washing in PBS. BrdU was detected by incubation for 10 min with the chromagen 3,3′-diaminobenzidine. Sections were then washed in deionized water. Peroxidase activity was quenched for a second time with 3% H_2_O_2_ in PBS, followed by washings with water. Sections were blocked with casein and then incubated overnight at 4°C with FNDC3B rabbit polyclonal antibody (1:50 dilution), washed, and then incubated with horseradish peroxidase-conjugated broad spectrum secondary antibody. Sections were washed with PBS and FNDC3B staining was visualized after incubation with a contrast purple chromogen, ImmPACT™ VIP peroxidase substrate (Vector Labs). Sections were washed in deionized water, counterstained with 0.5% aqueous methyl green (Vector Labs), differentiated in 0.05% acetic acid/acetone, washed dehydrated in ethanol, cleared in xylene, and mounted in DPX (Sigma). Omission of primary antibodies was used for negative controls.

Immunofluorescence staining was performed, as described previously (Capuco, 2007), to determine the ESR1 status of LREC by assessing the co-localization of BrdU and ESR1.

## Results

### Identification of LREC in the terminal ductular units of bovine mammary gland

During the period of ductal morphogenesis, the prepubertal mammary gland grows allometrically and mammary ducts expand into the surrounding mammary fat pad (Capuco et al., 2002; Meyer et al., 2006). The terminal ductular units of the prepubertal mammary gland, which are prevalent at this time, are arborescent structures composed of a multilayered epithelium (Capuco et al., 2002; Figures 1A,B). One approach, which we have utilized, to identify putative stem cells is based on the observation that somatic stem cells often retain labeled DNA strands for a prolonged period after initial labeling with tritiated thymidine or BrdU (Potten et al., 1978; Bickenbach, 1981). In mice, intestinal crypt cells (Potten et al., 2002), muscle satellite cells (Conboy et al., 2007), and putative MaSC (Welm et al., 2002; Smith, 2005) retain labeled DNA. Although long-term retention of BrdU does not appear to be a universal marker for somatic stem cells, it appears to provide a means for identifying putative stem/progenitor cells in mammary gland. After staining BrdU-labeled cells in cryosections without compromising RNA quality (Choudhary et al., 2010), we employed laser microdissection to collect LREC from basal and embedded layers of the mammary epithelium, along with appropriate control cells (Figures 1C,D). The transcriptome of these cells was interrogated by microarray analysis, from which we based our characterization of these interesting LREC in bovine mammary gland.

### Transcriptomes of LRECb vs. ECb

To evaluate the hypothetical stem cell nature of LRECb, we compared the transcript profiles of LRECb vs. neighboring control cells (ECb). This analysis identified 605 genes that were differentially expressed between these two cell types (Table S1 in Supplementary Material). Of these, 476 corresponded to genes that were functionally annotated in the Ingenuity Pathway Analysis database. Differentially expressed genes were involved in pathways linked to cancer, gene expression, cell growth and proliferation, and cell death (Table S2 in Supplementary Material). A number of genes with documented relevance to MaSC were identified in this analysis (Tables 1 and 2). Low expression of *ESR1* and high expression of ALDH 3B1 (*ALDH3B1*) in LRECb were consistent with MaSC character. Similar to the situation in mouse and human, putative bovine MaSC (LRECb) appear to be ESR1-negative (Capuco et al., 2009; Figures 7E,F), and increased ALDH activity is consistent with MaSC/progenitor character (Douville et al., 2009; Martignani et al., 2010; Rauner and Barash, 2012). Increased abundance of *HNF4A*, *NR5A2*, *NES*, *TERF1*, *NUP153*, and *FNDC3B* mRNA and decreased abundance of X-chromosome inactivation factor *(XIST*) in LRECb are noteworthy (Table 1 and Table S1 in Supplementary Material). Hepatocyte nuclear factor (HNF4A) is a liver stem cell transcription factor (Battle et al., 2006; Delaforest et al., 2011), NR5A2 is a pluripotency transcription factor analogous to OCT4 (Heng et al., 2010), Nestin (NES) is a neural stem cell marker (Wiese et al., 2004), and TERF1 (Telomeric repeat binding factor 1) is a marker for human and mouse embryonic stem cells (Ginis et al., 2004). *FNDC3B* has been characterized as a marker of proliferation and cell migration. The absence or very low abundance of *XIST*, in LRECb is consistent with MaSC identity, as absence of *XIST* expression and low *XIST* expression have been associated with hematopoietic stem and progenitor cells, respectively (Savarese et al., 2006). Transcripts of several genes that are involved in epigenetic modification of chromatin were also enriched in LRECb. Relative to ECb, LRECb expressed a greater number of transcription regulators, zinc fingers, and nuclear transporters (e.g., *NUP153, IPO13*). Importin 13 (*IPO13*) is a nucleocytoplasmic transport protein, which may serve as a marker for corneal epithelial progenitor cells (Wang et al., 2009). Because elements of the nuclear pore complex and importin are frequently down-regulated following cell differentiation (Yasuhara et al., 2009), increased expression of *NUP153* and *IPO13* in LRECb suggests that LRECb are undifferentiated epithelial cells. Recent research by Sherley and colleagues was undertaken to discover biomarkers for distributed stem cells, based upon identification of genes that are tightly coupled to asymmetric self renewal of cells in culture (Noh et al., 2011). Among the genes identified by these researchers, expression of *EPHX1*, *MTBP*, *COL11A1*, and *ARHGAP* was increased in LRECb in the current experiment. Finally, expression of cytokeratin markers was consistent with expression by MaSC. The basal epithelial cells were KRT19-negative (Figure 1E), and transcriptome analysis indicated that *KRT5* was strongly down-regulated in LRECb, consistent with MaSC (Petersen and Polyak, 2010). Transcripts for fibroblast growth factors (*FGF1*, *FGF2*, *FGF10)*, insulin-like growth factor-2 *(IGF2)* and follistatin *(FST)* were also enriched in LRECb. Overall, the gene expression profile of LRECb is consistent with MaSC character (Tables 1 and 2).

**Table 1:** **Attributes of LREC in prepubertal bovine mammary epithelium^1^**.

Gene	LRECb LRECe	Description
**STEMNESS MARKERS**		
**Transcription Factors**		
*NR5A2*	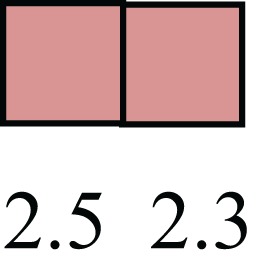	Pluripotency transcription factor can substitute for OCT4 in production of induced pluripotent stem cells (iPSC)
*HNF4A*	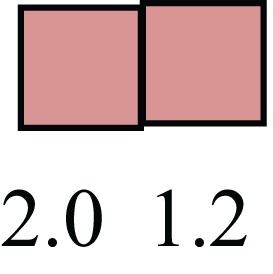	Liver stem cell transcription factor
*SOX15*	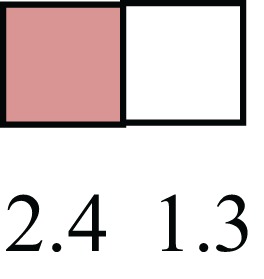	Encodes a member of the SOX (SRY-related HMG-box) family of transcription factors involved in the regulation of embryonic development and in the determination of the cell fate
*ESR1*	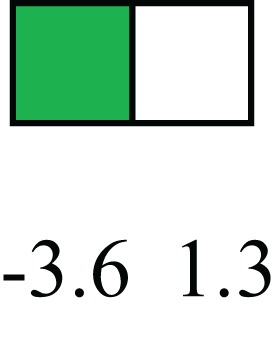	LRECb are ESR1-negative (immunohistochemistry) and express low levels of ESR1 transcripts. ESR1 is a negative marker for MaSC in mouse and human. LRECe are ESR1-positive or negative
**Nuclear complex, pore, or transport**		
*NUP153*	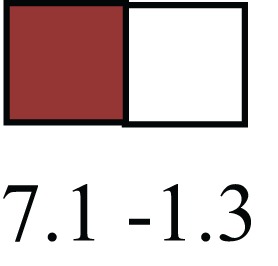	Nuclear basket protein, can cause chromatin modification, may be marker of proliferation, nuclear pore complex proteins are often down-regulated in differentiated cells
*IP013*	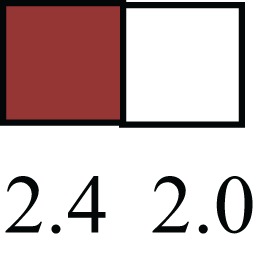	Nucleocytoplasmic transport protein, importin 13, may serve as a marker for corneal stem and progenitor cells, nuclear transport plays a key role in stem cell lineage determination
*TERF1*	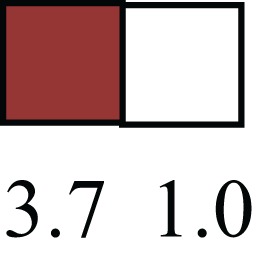	Telomeric repeat binding factor 1, marker for human and mouse embryonic stem cells
**Cytoskeleton or membrane associated**		
*THY1*	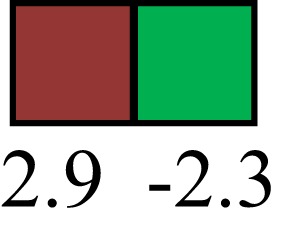	THY1/CD90 is up-regulated in MaSC enriched human mammary epithelial cultures [Expression of *THY1* in LRECb did not differ from that in ECb, but was 5.6-fold greater than that in LRECe]
*HIP1*	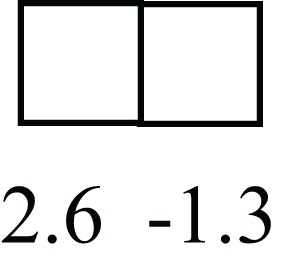	May be required for differentiation or survival of somatic progenitors [Expression of *HIP1* in LRECb did not differ from that in ECb, but was 3.1-fold greater than that in LRECe]
*NES*	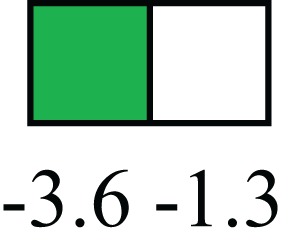	Intermediate filament protein, expressed in stem cells: neural and hair follicle. [Expression of *NES* in LRECb did not differ from that in ECe, but was 2.6-fold greater than that in LRECe]
*FNDC3B*	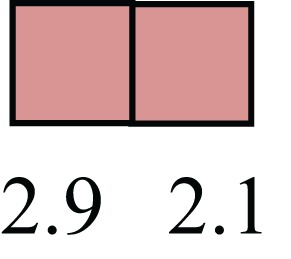	Regulator of adipogenesis and cell proliferation, adhesion, spreading, and migration
**Enzyme or Kinase**		
*ALDH3B1*	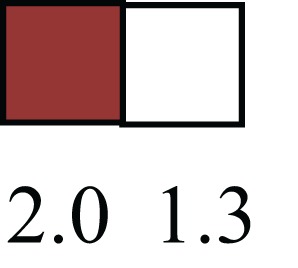	Proposed MaSC marker
*TRIB2*	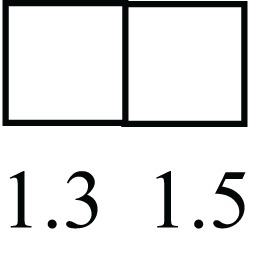	Modulates signal transduction pathways and may promote growth of mouse myeloid progenitors. [Expression of TRIB2 in LRECb did not differ from that in ECb, but was 6.7-fold greater than that in LRECe]
**Asymmetric cell renewal**		
*EPHX1, MTBP, COL11A1*	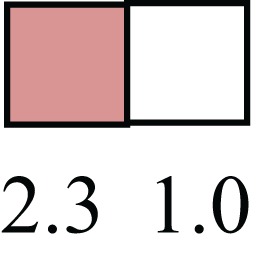	Among proposed markers for somatic stem cells, based on genes that are tightly coupled to asymmetric self-renewal *in vitro* (Noh et al., 2011)
*ARHGAP*	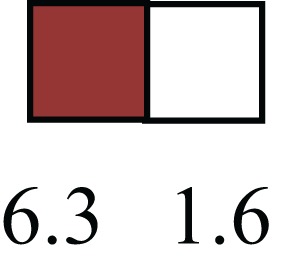	
**DIFFERENTIATION MARKERS**		
**Transcript Factor**		
*XIST*	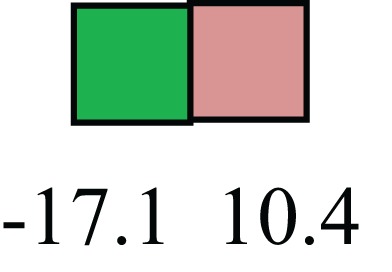	No to little expression of X-chromosome inactivation factor in LRECb, absence or low expression has been associated with hematopoietic stem and progenitor cells, respectively
*SIX2*	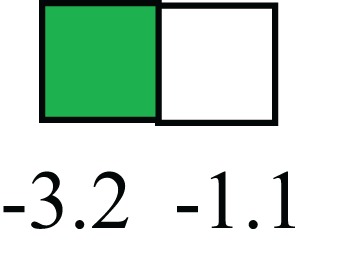	Down-regulated in LRECb, is a homeobox protein, transcription factor. Like other members of this family, it may be involved in differentiation, involved in limb or eye development
*PAX6*	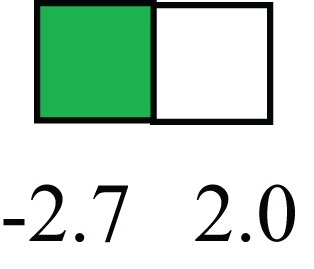	Down-regulated in LRECb, is a paired box protein transcription factor. Implicated role in organogenesis. Possible role of PAX6 in breast cancer and tumorigenesis has recently been identified
**Intermediate Filaments**		
*KRT5*	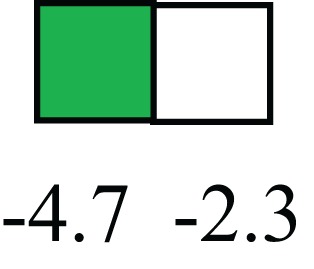	Cytokeratin 5 transcripts are down-regulated in LRECb, consistent with KRT5-negative MaSC in mouse and human
*KRT19*	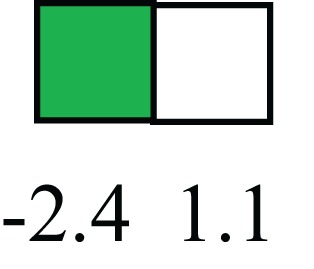	The basal epithelium is typically KRT 19 negative, consistent with KRT19-negative MaSC in mouse and human. [Expression based on quantitative immunohistochemistry]

*^1^The transcript abundance in LRECb and LRECe are expressed relative to that in respective control cells. Abundance that varies significantly in LREC and control cells is depicted graphically, with the fold change provided below the graphic. Fold change is provided even for those genes whose abundance did not differ between the LREC class and its control cells (designated by open bar)*.

*
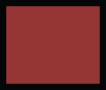
 Transcripts were up-regulated greater than threefold change relative to respective control*.

*
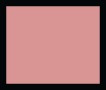
 Transcripts were up-regulated greater than two but less than threefold change relative to respective control*.

*
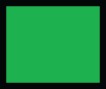
 Transcripts were down-regulated greater than twofold change relative to respective control*.

*
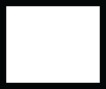
 Transcripts abundance did not differ from respective control*.

**Table 2:** **Pathways in LREC and microenvironment of the basal mammary epithelium**.

**PATHWAY INVOLVEMENT WITH LREC**	
Notch pathway	Up-regulation of Notch pathway in LRECb. Involvement of Notch pathway in LRECb along with pathways promoting expansion of MaSC is consistent with the need for balanced expansion of both MaSC and progenitor cell populations. Both populations must expand during ductal elongation
TGF-β signaling	Up-regulation in LRECb of components of TGF-β pathway associated with stem cell renewal
MAPK pathway	Up-regulation in LRECb, pathway involved in cell growth and proliferation
WNT pathway	Involvement of WNT pathway. Up-regulation of many components in LRECb. Presumed activity in LRECe > LRECb
**MICROENVIRONMENT OF BASAL MAMMARY EPITHELIUM**	
LRECb	Fibroblast growth factors (*FGF2, FGF10*), insulin-like growth factor-2 (*IGF2*), follistatin (*FST*), cytokines (*IL33, CSF3*), Wnt chaperone (*MESDC2*), heat inducible factors (*HIF1A, HSP*)
ECb	Production of factors by ECb that may impact LRECb: *JAG-1* (a ligand of Notch pathway) fibroblast growth factors (*FGF1, FGF2, FGF10*), insulin-like factor-2 (*IGF2*), follistatin like 1 (*FSTL1*), extracellular matrix and its regulators (collagens, *ITGB1*, *MFAP5, FBN1, FSTL1, CHAD, ERBB2IP, SPARC*), and tumor suppressors (*MTSS1*, Myc binding proteins)
Stromal cells	Gene expression by stromal cells was not assessed. However, these cells also contribute to the microenvironment of the basal epithelium

**Figure 2:**
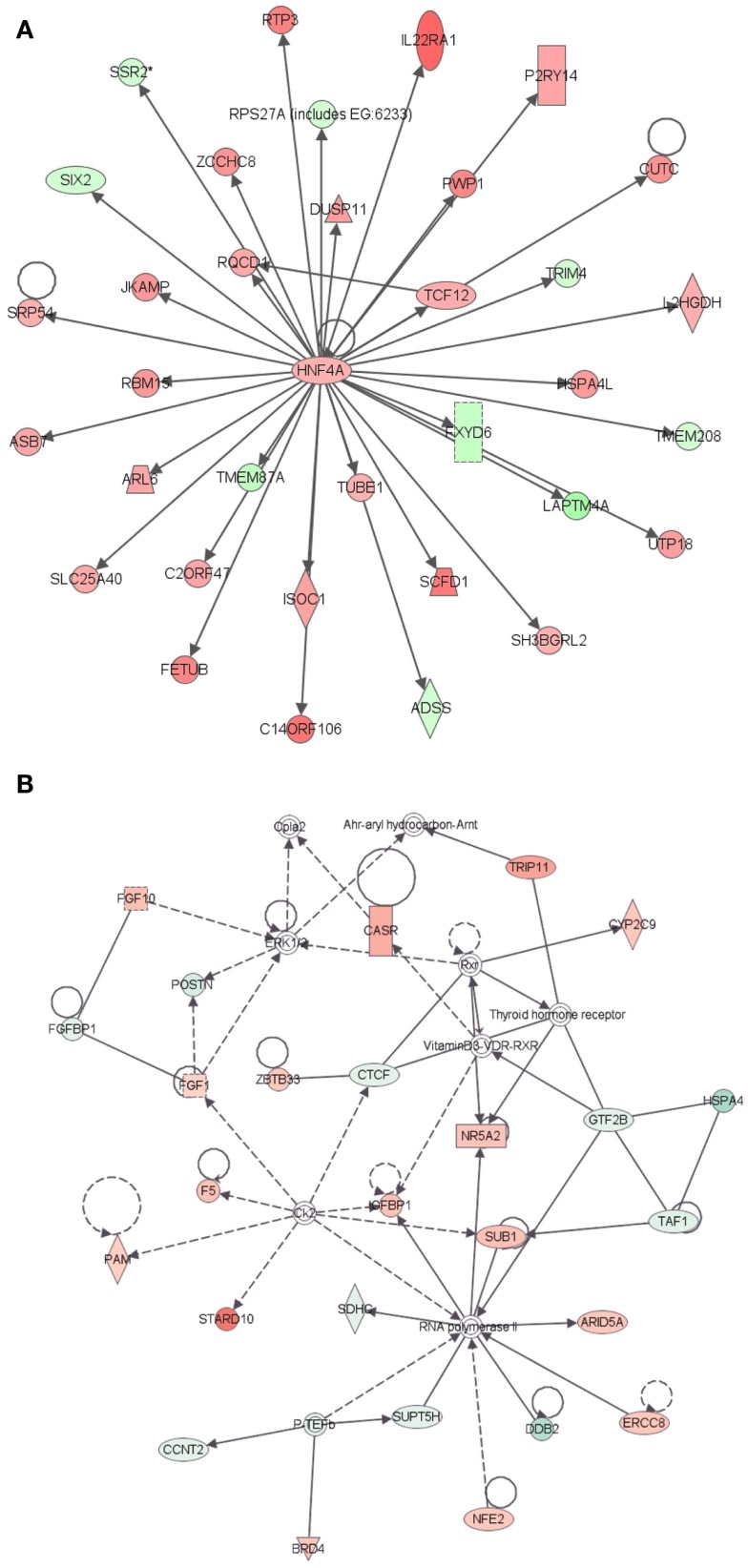
**Ingenuity Pathway Analysis (IPA) of genes differentially expressed in LRECb vs. ECb**. Genes that were differentially expressed in LRECb vs. ECb were imported into IPA software, which revealed the involvement of several networks pertinent to LRECb. Network **(A)** pertains to cellular growth and proliferation and shows a single module with *HNF4A* at its hub. Network **(B)** relates to cell cycle and post translational modification. Red color denotes up-regulation in LRECb and green color denotes down-regulation in LRECb relative to control cells. The IPA legend is shown in Figure A1 in Appendix.

Further evidence in support of the stem cell nature of LRECb comes from biological pathway analysis of differentially expressed genes. Ingenuity Pathway Analysis of genes that were differentially expressed in LRECb and ECb revealed biological processes and networks that were highly significant. (Significance of a biologically relevant network of genes was expressed in IPA score, which was derived from *P*-value and indicates likelihood of the focused genes in a network being found together due to random chance. The IPA score is expressed as the negative log of the *P*-value.) The most significant networks associated with LRECb related to cellular growth and proliferation (Figure 2A, IPA score = 58), and cell cycle and post translational modification (Figure 2B, IPA score = 34). The network of cellular growth and proliferation (Figure 2A) contains a single module with *HNF4A*, up-regulated in LRECb, as the hub. Down-regulation of developmental genes like *SIX2* and *XIST* suggests that LRECb are undifferentiated cells. KEGG pathway analysis using DAVID (Huang da et al., 2009) revealed that genes which were differentially expressed in LRECb vs. ECb reflected up-regulation of several pathways. These included the MAPK pathway (*FGF1, FGF2,FGF10, TAOK3, BRAF, ATF4, CREB, HSPA8, PDGFB, CDC25B*), a pathway involved in cellular growth and proliferation, and the WNT (*DVL2, PPP2R5E, SMAD4*) and TGF-β (*FST* and *SMAD4*) pathways, which are associated with stem cell renewal (Esmailpour and Huang, 2008; Mazumdar et al., 2010). In contrast to other members of the WNT pathway, *HOXA9* was strongly down-regulated in LRECb.

### Transcriptomes of LRECe vs. ECe

**Figure 3:**
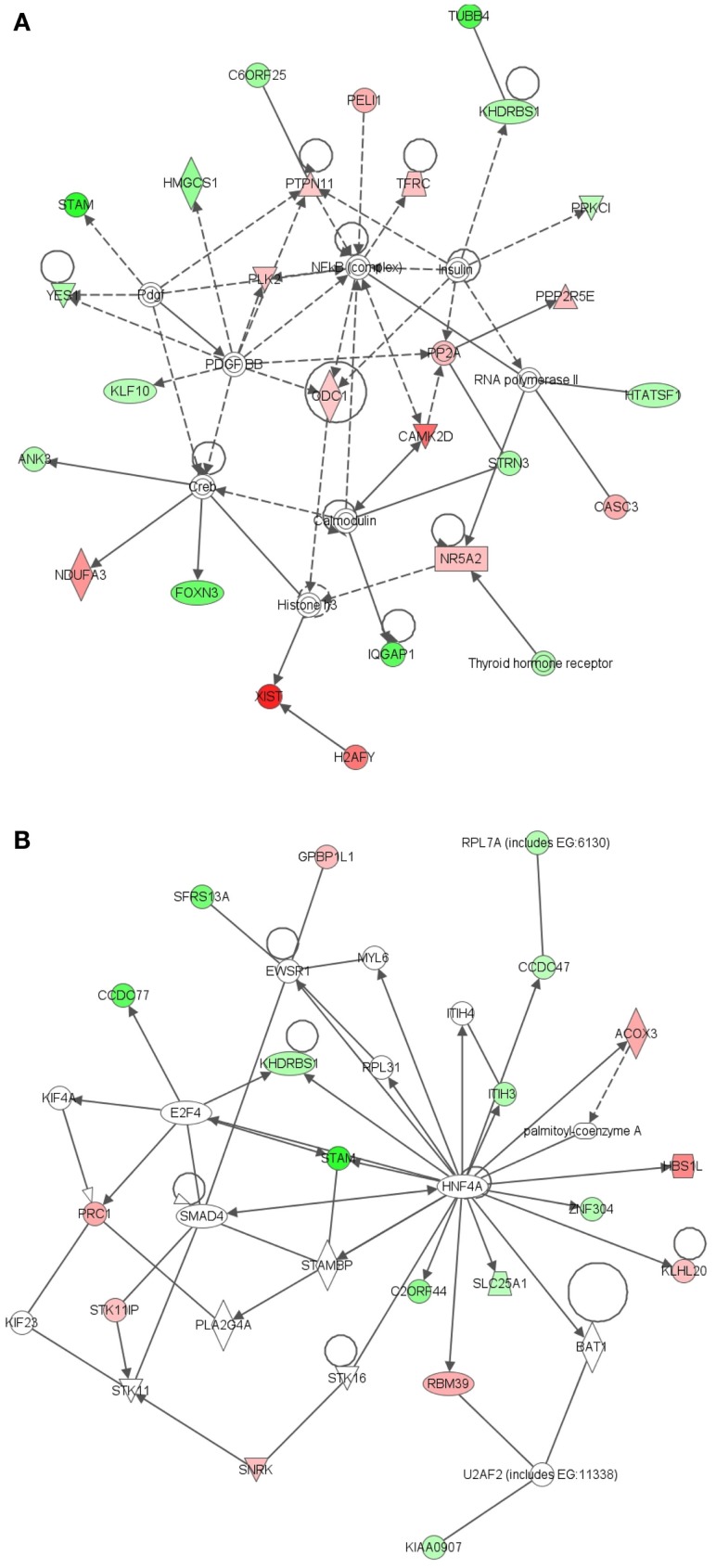
**Ingenuity Pathway Analysis (IPA) of genes differentially expressed in LRECe vs. ECe**. Genes that were differentially expressed in LRECe vs. ECe were imported into IPA software, which revealed the involvement of several networks pertinent to LRECb. Network **(A)** relates to cancer. Network **(B)** pertains to DNA replication, recombination and repair and contains a *HNF4A* module. Red color denotes up-regulation in LRECe and green color denotes down-regulation in LRECe relative to control cells. The IPA legend is shown in Figure A1 in Appendix.

Comparison of transcriptome profiles of LRECe and neighboring ECe identified 101 functionally annotated genes that were differentially expressed (Table S1 in Supplementary Material) and supports classification of LRECe as progenitor cells (Table 1). The most significant network associated with these genes was related to cancer (Figure 3A, IPA score = 51), followed by a network associated with DNA replication, recombination and repair (Figure 3B, IPA score = 36) that contained a *HNF4A* module. Conservation of the *HNF4A* module in LRECe and LRECb suggests a hierarchical similarity between LRECe and LRECb; although *HNF4A* transcripts were not significantly up-regulated in LRECe and genes involved in this module differed between the two categories of LREC. Enriched expression of *NR5A2* and *FNDC3B* in both LRECe and LRECb (vs. ECe and ECb, respectively) provides another line of evidence for the similarity of LREC in basal and embedded epithelial layers. KEGG pathway analysis (DAVID) of transcripts that were up-regulated in LRECe vs. ECe identified up-regulation of the WNT pathway (*DVL3, ADCY6, CAMK2D*) and down-regulation of an inhibitor of the WNT pathway, (*CAMK2N1*).

### Transcriptomes of LRECb vs. LRECe

To evaluate the relative characteristics of LRECb and LRECe, transcript profiles for these cells were compared. We identified 269 genes that were differentially expressed in LRECb vs. LRECe (Table S1 in Supplementary Material). Relatively high expression of stem cell markers, growth, and survival factors, DNA repair enzymes and low expression of apoptotic genes and differentiation markers supported greater “stemness” of LRECb vs. LRECe (Tables 1– 3). The molecular profile of LRECb included increased abundance of transcripts for stem cell markers (*NR5A2, NES, THY1*), as well as cell survival and proliferation factors (*IGF2, FGF2, FGF10, HSPB6*, *LAMC1, CSF3, FST, IL33, MESDC2, AGT*). Additionally, enriched expression of cell adhesion molecules (*CADM3, NCAM1, AOC3*), and a number of cell surface markers *(ANXA6, CCR1, CCR4, CXCR4, DRD2, GNB4, GRB14, SAT2, SDPR, THY1/CD90, TRIB2)* were noted in LRECb. THY1 has been used as a marker for hematopoietic progenitor cells (Goldschneider et al., 1978; Craig et al., 1993), mesenchymal stem cells (Gargett et al., 2009), and mammary cancer stem cells (Diehn et al., 2009). The LRECe displayed increased expression of *XIST*, splicing factor arginine/serine-rich 5 (*SFRS5*), *THAP* domain containing apoptosis associated protein 3 (*THAP3*), and calcium/calmodulin-dependent protein kinase II delta (*CAMK2D*). Increased expression of glucose metabolic enzymes, glucose phosphate isomerase (*GPI*), and UDP-glucose pyrophosphorylase 2 (*UGP2*) was also evident in LRECe vs. LRECb. KEGG pathway analysis (DAVID) revealed up-regulation of the Notch pathway in LRECb (*DVL2* an inhibitor of the pathway is down-regulated, *CAMK2D* and *MAML3* are up-regulated).

**Table 3:** **Top 10 up-regulated and down-regulated genes in LRECb vs. LRECe**.

Gene symbol	Bovine RefSeq ID	Gene ID	Gene annotation	Fold change
**UP-REGULATED IN LRECb**				
*LOC786372*	XM_001254067.2	297472984	PREDICTED: *Bos taurus* uncharacterized	14.3
*RPS7*	NM_001098874	402744265	*Bos taurus* ribosomal protein S7	12.5
*CFL1*	NM_001015655.1	62751776	*Bos taurus* cofilin 1 (non-muscle)	8.3
*RRN3*	NM_001192432	329112519	*Bos taurus* RRN3 RNA polymerase I transcription factor homolog (*S. cerevisiae*)	7.1
*COL1A2*	NM_174520.2	31341767	*Bos taurus* collagen, type I, alpha 2	7.1
*TRIB2*	NM_178317.3	58332431	*Bos taurus* tribbles homolog 2 (*Drosophila*)	6.7
*HSPB6*	NM_001076027.1	115496723	*Bos taurus* heat shock protein, alpha-crystallin-related, B6	6.7
*NUP153*	NM_001205754	329664715	*Bos taurus* nucleoporin 153kDa	6.3
*SLC10A5*	XM_001253131.2	194672838	PREDICTED: *Bos taurus* similar to solute carrier family 10 (sodium/bile acid cotransporter family), member 5	5.9
*RPL13*	NM_001015543	402692502	*Bos taurus* ribosomal protein L13	5.9
**DOWN-REGULATED IN LRECb**				
*XIST*	NR_001464.2	166706871	*Bos taurus* X (inactive)-specific transcript, non-coding RNA	−5.6
*C19H17orf49 (BAP18)*	NM_001038092.1	84000150	*Bos taurus* chromosome 17 open reading frame 49 ortholog	−3.9
*SFRS5*	NM_001098929.1	149643058	*Bos taurus* splicing factor, arginine/serine-rich 5	−3.7
*THAP3*	NM_001075347.1	115496479	*Bos taurus* THAP domain containing, apoptosis associated protein 3	−3.7
*STK11IP*	XM_593410	358411062	PREDICTED: *Bos taurus* similar to LKB1 interacting protein	−3.7
*UGP2*	NM_174212.2	110347575	*Bos taurus* UDP-glucose pyrophosphorylase 2	−3.4
*SLC3A2*	NM_001024488.2	164448599	*Bos taurus* solute carrier family 3 (activators of dibasic and neutral amino acid transport), member 2	−3.3
*CAMK2D*	NM_001046333.1	114052473	*Bos taurus* calcium/calmodulin-dependent protein kinase II delta	−3.3
*EIF4E3*	NM_001102306.1	156121256	*Bos taurus* eukaryotic translation initiation factor 4E family member 3	−3.3
*ATXN7L3B*	NM_001078161.2	210147444	*Bos taurus* ataxin 7-like 3B	−3.0

**Figure 4:**
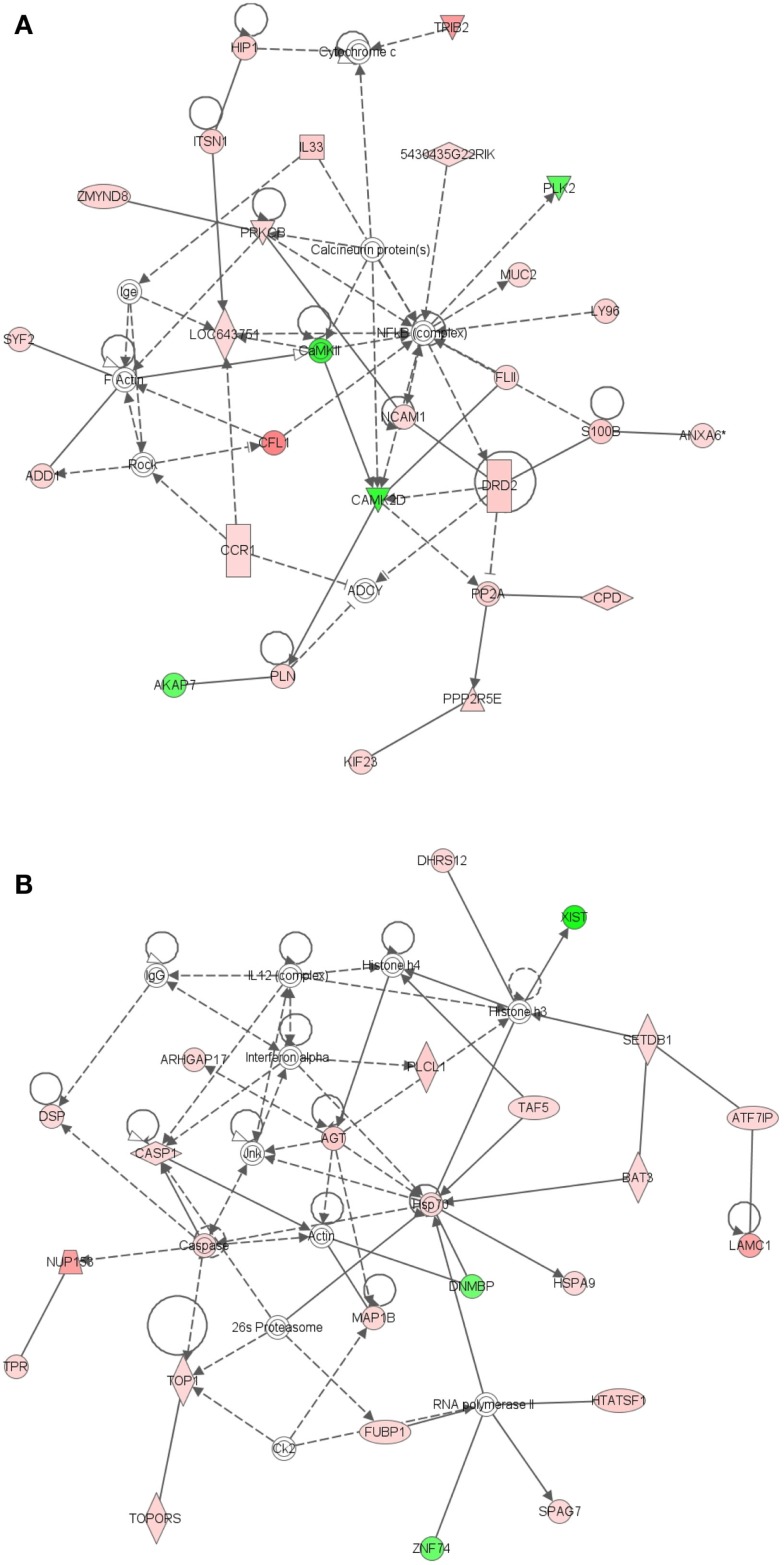
**Ingenuity Pathway Analysis (IPA) of genes differentially expressed in LRECb vs. LRECe**. Genes that were differentially expressed in LRECb vs. LRECe were imported into IPA software, which revealed the involvement of several networks pertinent to LRECb. Network **(A)** pertains to tissue development, cell growth and proliferation. Network **(B)** is associated with tissue injury and contains a heat shock protein module that was up-regulated in LRECb. Red color denotes up-regulation in LRECb and green color denotes down-regulation in LRECb relative to control cells. The IPA legend is shown in Figure A1 in Appendix.

The most significant network associated with genes that were differentially expressed in LRECb vs. LRECe was related to tissue development, cell growth, and proliferation (Figure 4A, IPA score = 43). This network showed up-regulation in LRECb of *HIP1*, which may be required for differentiation or survival of somatic progenitors, and *TRIB2*, which modulates signal transduction pathways and may promote growth of mouse myeloid progenitors. This was followed by a network associated with tissue injury (Figure 4B, IPA score = 34), featuring up-regulation of a heat shock protein module in LRECb. The top three canonical pathways identified by IPA for genes that were preferentially expressed by LRECb (LRECb vs. LRECe) pertained to: the mitotic roles of polo-like kinases, cleavage, and polyadenylation of pre-mRNA, and chemokine signaling. Because polo-like kinases are key centrosome regulators and asymmetric localization of polo-kinase promotes asymmetric division of adult stem cells (Rusan and Peifer, 2007), the polo-like kinase pathway may be particularly noteworthy.

### Transcriptomes of ECb vs. ECe

Epithelial cells isolated from basal and embedded layers exhibited transcriptome profiles that were consistent with their location. Analysis identified 317 genes that were differentially expressed (Table S1 in Supplementary Material), 263 of which were functionally annotated. Among these, ECb expressed increased transcript levels for cell structural and motility genes, including actin (*ACTA2*), myosin (*MYH8, MYO6, MRCL3*), *SPTBN1* (actin cross linking scaffold protein), and *TSPAN31*. Transcripts for *JAG-1* (ligand of Notch pathway) and FST like 1 (*FSTL1*) were enriched in basal epithelium. The enriched expression of integrin-β1 (*ITGB1*) within ECb was consistent with its use as a marker to isolate MaSC (Shackleton et al., 2006), most likely to enrich the sorted population for basal epithelial cells. Additionally, a number of heat shock proteins (*HSPA8, HSPA4, HSP90AB1*), peptidases (*USP4, USP16, USP25, PSMD14, MME*), ribosomal proteins, translational regulators, components of the ECM and its regulators (*collagens, MFAP5, FBN1, FSTL1, CHAD, ERBB2IP, SPARC*), and tumor suppressors [*MYCBP2*, and *MTSS1* (LOC788499)] were also up-regulated in ECb. However, transcripts of membrane transporters (*AP1M1, APOE, AQP7, SLC13A3, SLC38A3, TMED3*, *CLCN3*) were more highly expressed in ECe than ECb. Thus, control cells harvested from basal and from embedded layers within the mammary epithelium possess different characteristics and appear to represent two distinct cell populations.

**Figure 5:**
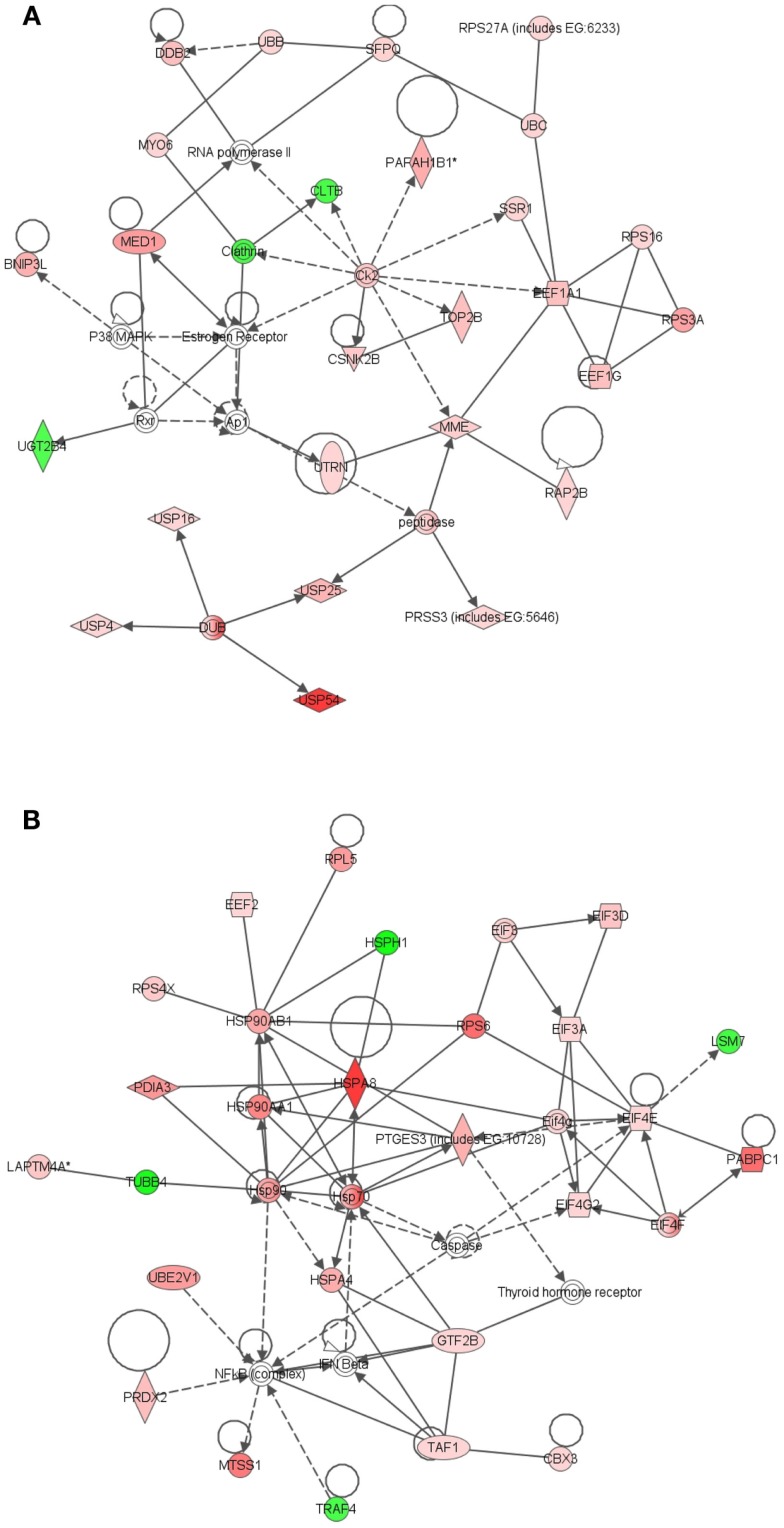
**Ingenuity Pathway Analysis (IPA) of genes differentially expressed in ECb vs. ECe**. Genes that were differentially expressed in ECb vs. ECe were imported into IPA software, which revealed the involvement of several networks pertinent to ECb. Network **(A)** pertains to endocrine development and function, lipid metabolism. Network **(B)** is associated with cancer and contains two heat shock protein modules. Red color denotes up-regulation in LRECb and green color denotes down-regulation in LRECb relative to control cells. The IPA legend is shown in Figure A1 in Appendix.

**Figure 6:**
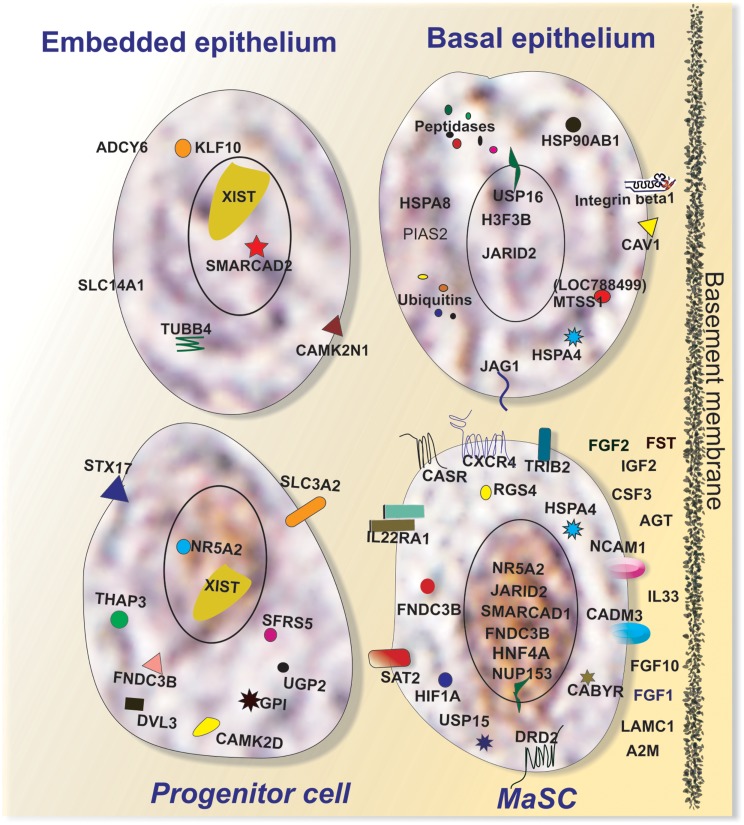
**Characteristics of putative MaSC (LRECb) and progenitor cells (LRECe)**. Putative MaSC (LRECb) are localized in the basal epithelium in a stem cell niche characterized as hypoxic but an environment enriched for extracellular growth factors, tumor suppressors for regulating MaSC function. LRECb exhibit enriched expressions of adhesion molecules and a variety of potential MaSC biomarkers including HNF4A and the pluripotency marker, NR5A2. Putative progenitor cells (LRECe) also express NR5A2 but at a reduced level along with increased expression of differentiation factors including XIST. Key features of control cells (non-LREC) in the basal and embedded epithelial layers are also depicted.

To better understand key biological processes occurring in basal and embedded epithelium, we utilized Ingenuity Pathway Analysis to generate gene networks and canonical pathways for genes that are differentially expressed between ECb and ECe. All identified networks (networks of endocrine system development and function, cancer, cell cycle, tissue development) were highly significant as measured by IPA score (ranges from 35 to 42). The identified network for endocrine development and function, lipid metabolism (Figure 5A) features an estrogen signaling module, peptidase, Ubiquitination, and ubiquitin modules. The identified network for cancer (Figure 5B) contains two heat shock protein modules. The canonical pathways identified by IPA analysis were protein ubiquitination, hypoxia signaling, and clathrin mediated endocytosis. Extrinsic growth factors and regulators, and hypoxia inducing factor have been identified as molecules prevalent in the stem cell niche (Li and Xie, 2005; Mazumdar et al., 2010), transcripts for these molecules are expressed in the basal epithelium (Table 2; Figure 6).

### Immunohistochemical and realtime RT-PCR evaluation of potential novel LRECb and LRECe markers

Genes that are highly expressed in LRECb and LRECe may provide novel markers for MaSC and progenitor cells. Those that were evaluated by immunohistochemistry were: *NR5A2*, *NUP153, FNDC3B*, and *HNF4A*. *NR5A2* is a pluripotency gene that aids in inducing somatic cells into pluripotency (iPSC; Heng et al., 2010). *NUP153* is a nuclear basket protein that can cause chromatin modification (Vaquerizas et al., 2010), and *FNDC3B* is a regulator of adipogenesis and cell proliferation, adhesion, spreading, and migration (Nishizuka et al., 2009). *HNF4A* may serve as a stem cell regulator (Battle et al., 2006; Koh et al., 2010; Delaforest et al., 2011) and was identified as a key pathway component by IPA analysis of expression data for LRECb and LRECe. Transcripts for *NR5A2, NUP153*, *FNDC3B*, and *HNF4A* were more abundant in LRECb than in control cells, with a general expression pattern of LRECb > LRECe > EC).

**Figure 7:**
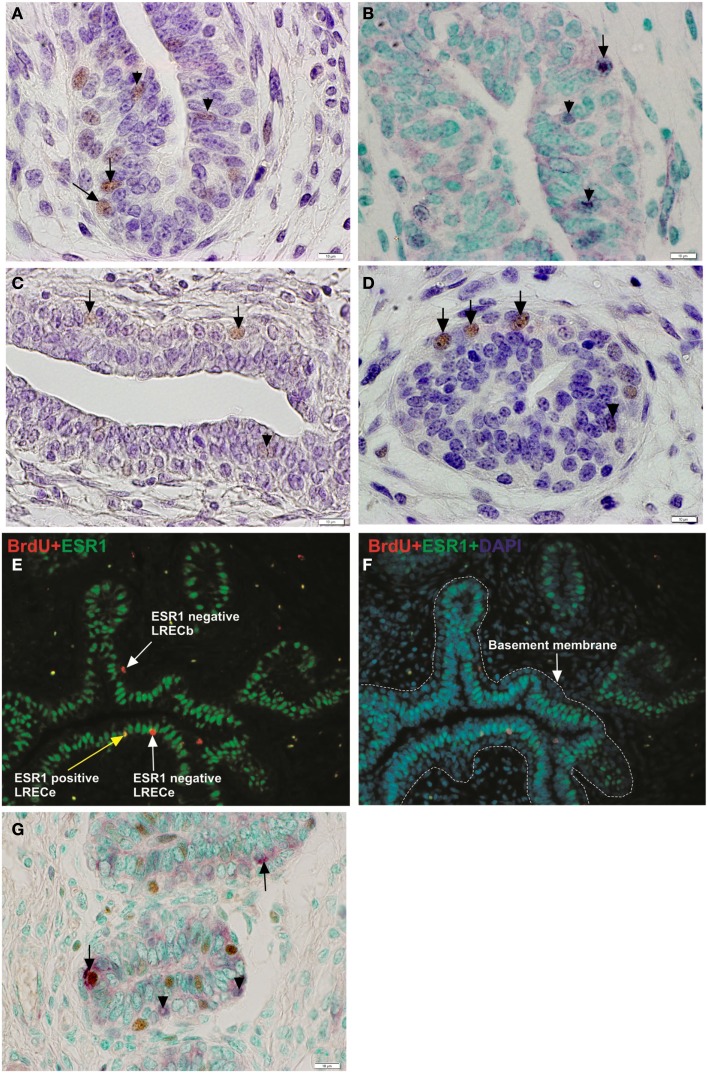
**Immunohistochemical localization of potential mammary stem/progenitor cell markers**. **(A–D)** Consistent with transcriptome data, cells that were positive for these novel markers were more strongly labeled (brown nuclei) in the basal layer of mammary epithelium than in the embedded layers. Solid arrows designate labeled nuclei of basal cells and arrow heads designate labeled nuclei of embedded epithelial cells. NR5A2 **(A)**, NUP153 **(B)**, FNDC3B **(C)**, HNF4A **(D)**. **(E,F)** Immunofluorescent micrograph demonstrating the ESR1 status of LREC. LRECb were ESR1-negative and LRECe were a mixed population of ESR1-negative and ESR1-positive cells. **(G)** Co-localization of FNDC3B (purple) and BrdU. FNDC3B was expressed in the nucleus (arrow) and cytoplasm (arrowhead) of LRECb (brown). Scale bar, 10 μm.

Immunohistochemical analysis showed that 1–6% of epithelial cells expressed these potential markers. In agreement with transcript abundance, positive cells in the basal epithelium were more intensely stained than those in suprabasal locations. The abundance and localization of NR5A2, NUP153, FNDC3B, and HNF4A-positive cells (Figures 7A–D) were similar to that of LRECs. Co-localization studies showed that LREC expressed these markers. Surprisingly, expression of FNDC3B was not limited to the cytoplasmic compartment of the cell. Expression of FNDC3B was found to be cytoplasmic (arrows) and nuclear (arrowheads) and co-expressed with BrdU in approximately half of the LRECb (Figure 7G), which is consistent with its possible utility as a marker for putative MaSC/progenitor cells. Co-localization studies also confirmed our previous finding (Capuco, 2007; Capuco et al., 2009) that LRECb are ESR1-negative and LRECe are composed of populations of ESR1-negative and ESR1-positive cells (Figures 7E,F). Because of their potential utility for cell sorting, we also identified transcripts that encoded surface proteins and were up-regulated in LRECb (*SAT2, CXCR4, SDPR, RTP3, CASR, GNB4*, *and DRD2)*; however, we have not evaluated the suitability of these membrane markers. Preliminary immunohistochemistry results showed that CXCR4 and CASR are expressed by a small number of epithelial cells.

**Figure 8:**
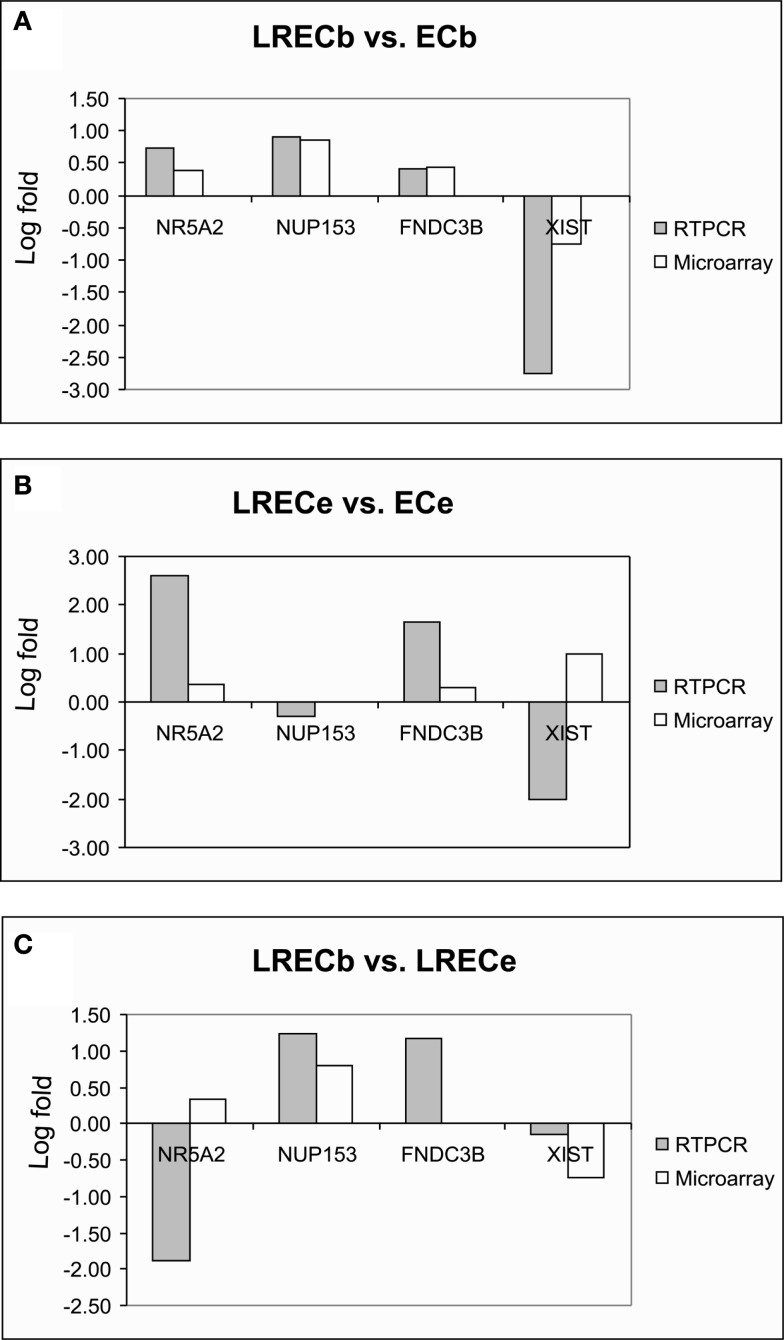
**Quantitative RT-PCR and microarray analysis of selected genes**. Concordance of gene expression patterns between microarray and qRT-PCR for key genes. **(A)** LRECb vs. ECb; **(B)** LRECe vs. ECe; **(C)** LRECb vs. LRECe. Data are expressed as fold change (log_10_) relative to indicated comparisons.

Realtime RT-PCR was employed to confirm microarray results for expression of transcripts for novel LREC-derived markers (*NR5A2, NUP153, FNDC3B*) and the differentiation factor *XIST* at the transcriptome level. Patterns of expression were very similar for RT-PCR and microarray analysis (Figures 8A–C). Both analyses showed that expression of the potential MaSC/progenitor cell markers was increased in LRECb and, with the exception of *NUP153*, in LRECe vs. their respective controls. Expression of these markers was greater in LRECb vs. LRECe by microarray analysis, but *NR5A2* expression was not greater in LRECb vs. LRECe when assessed by realtime RT-PCR. Consistent with the undifferentiated state of putative MaSC, there was little to no expression of the differentiation factor *XIST* in LRECb, and there was lower expression of *XIST* in LRECb than in LRECe by both methodologies. Expression of *XIST* non-coding RNA was less in LRECe than in control cells as assessed by RT-PCR, but greater when assessed by microarray hybridization. Overall, the utility of microarray data for detecting LREC-derived markers for putative MaSC/progenitor cells was supported by realtime RT-PCR and by immunohistochemistry.

## Discussion

In this study, we employed the long-term retention of BrdU-labeled DNA to identify putative MaSC/progenitor cells during the period of ductal morphogenesis in the prepubertal mammary gland. However, it must be understood that retention of labeled DNA represents an integration of a cell’s past proliferation and differentiation events and may not reflect that cell’s current status. This is particularly relevant when assessing individual cells within a population, e.g., expression of lineage markers by LREC. Nonetheless, we hypothesized that LREC are enriched for MaSC/progenitor cells. In particular, we hypothesized that LRECb are enriched for MaSC and LRECe are enriched for progenitor cells.

When comparing gene expression in LREC and control cells it is important to consider the proliferative status of these cells. A difference in the proliferative status of the two populations may impose differences in gene expression between the populations that are reflective of their relative cell cycle activity rather than cell lineage. To determine the extent to which LREC proliferate during ductal morphogenesis in the prepubertal bovine mammary gland, we evaluated expression of nuclear proliferation antigens. In the present experiment, we found that approximately 13% of LREC and 15% of control cells in the present experiment expressed PCNA (data not shown). In previous studies, we evaluated the Ki-67 labeling index in calves at an equivalent stage of mammary development to those in the present experiment and reported that 5.4% of LREC expressed Ki-67 (Capuco, 2007) and that 5–8% of total epithelial cells expressed Ki-67 (Capuco et al., 2004). Thus, the proliferation status of LREC, control cells and the overall epithelial population appear to be similar and not likely to unduly influence interpretation of gene expression data.

To address our hypothesis that LRECb are enriched for MaSC and that LRECe are enriched for more committed progenitors, we performed transcriptome analyses on the four populations of bovine mammary epithelial cells obtained by laser microdissection of LREC and EC from basal and embedded layers of the epithelium. Microarray analysis was used to reveal gene signatures for the four categories of mammary epithelial cells: LRECb, LRECe, ECb, and ECe.

The ECb and ECe were distinguishable by the increased abundance, in basal cells, of transcripts for genes encoding structural and motility proteins, extracellular growth factors, extracellular matrix (ECM) proteins, and ECM regulators. Additionally, increased expression of transcripts for heat shock proteins, peptidases, ribosomal proteins, ubiquitins, proteins that provide interaction between the cell and the ECM (caveolin-1, integrin-beta-1), tumor suppressors, and epigenetic modifiers were also characteristic of ECb. Myoepithelial cells, present in the basal layer of mature mammary epithelium, may be a part of the stem cell niche and their paracrine factors may regulate the proliferation, polarity, and motility of mammary epithelial cells (Polyak and Hu, 2005). However, the precise nature of the ECb in a calf is uncertain. Expression of markers for myoepithelial cells in mammary tissue from prepubertal heifers is absent or expressed in a limited fashion (Capuco et al., 2002; Ballagh et al., 2008; Ellis et al., 2012; Safayi et al., 2012).

Transcriptome analysis of LRECb vs. ECb showed that LRECb possess characteristics consistent with those of MaSC (Tables 1 and 2). Our mRNA data indicated a reduced expression of *ESR1* and increased expression of *ALDH3B1* in LRECb vs. ECb, and immunohistochemistry demonstrated a lack of detectable ESR1 protein in LRECb. Previous studies have demonstrated that mouse (Sleeman et al., 2007) human (Anderson and Clarke, 2004) and putative bovine MaSC are ESR1-negative (Capuco et al., 2009). *ALDH1* activity has been used as a stem and progenitor cell marker in several tissues including blood, lung, prostate, pancreas, and breast (Douville et al., 2009). However, 17 isoforms of ALDH have been identified (Sladek, 2003) with different cellular and species expression patterns (Hess et al., 2004). *ALDH3B1* is expressed by bovine LRECb. Increased abundance of *HNF4A, NR5A2, TERF1, THY1, NUP153*, and *FNDC3B* mRNA and decreased abundance *XIST* transcripts (non-coding) in LRECb are noteworthy. *HNF4A* is a hepatic stem cell transcription factor whose associated network was highly up-regulated in LRECb, suggesting a key role in these cells. It is noteworthy that *HNF4A* has recently been implicated as a regulator of mesenchymal stem cells (Koh et al., 2010). Lack of expression or low expression of *XIST* has been associated with stem and progenitor cells, respectively, in hematopoietic tissue (Savarese et al., 2006). Subsequently, we evaluated four potentially novel protein markers for stem/progenitor cells (NR5A2, NUP153, FNDC3B, HNF4A) immunohistochemically and found protein expression profiles that were consistent with the observed transcript abundance in LRECb and LRECe. The number of cells expressing these markers was limited and staining intensity of the positive cells was greater for those located in the basal layer of the epithelium.

Because of their potential utility for cell sorting, we identified transcripts that encoded surface proteins and were up-regulated in LRECb. Among the cell surface markers, *THY1/CD90* is a proposed marker for mesenchymal, liver, keratinocyte, endometrial, and hematopoietic stem cells. *TRIB2* is an oncogene shown to prolong growth of mouse myeloid progenitors (Keeshan et al., 2006). *SAT2* is the target of DNA methyltransferase 1 (*DNMT1*) and an epigenetic modifier, whose methylation status may serve as a marker for cancer prognosis (Jackson et al., 2004). *CXCR4* is a receptor for the chemokine, stromal derived factor 1 (*SDF-1*; Kang et al., 2005). *SDF-1* is positively regulated by HIF1A, linking the SDF-CXCR4 axis to hypoxic stress. G-protein signaling proteins such as RGS4, which was up-regulated in LRECb, are negative regulators of the SDF-CXCR4 axis. The pertinence of the SDF-CXCR4 axis to stem cell regulation is the likelihood that mild hypoxic stress induces expansion of the MaSC population analogous to the expansion of breast cancer stem cells (Conley et al., 2012).

Up-regulation of growth factors such as fibroblast growth factors (*FGF1, FGF2, FGF10)*, insulin-like factor-2 *(IGF2)*, *FST*, laminin (LAMC2), platelet-derived growth factor beta (PDGFB), and plasminogen activator tissue (PLAT) in the basal epithelial layer is consistent with the possible function of these molecules as regulators of MaSC. The role of FGFs in mammary gland development and growth has been demonstrated (Mailleux et al., 2002; Sinowatz et al., 2006). Although our data do not provide evidence for enhanced expression of receptors for these growth factors in LRECb, transcripts for many of these receptors were evident.

Further evidence in support of LRECb being a population of cells that is enriched for MaSC comes from biological pathway analysis of differentially expressed genes. A number of differentially expressed genes (LRECb vs. ECb) were involved in MAPK, WNT, and TGF-β pathways. The MAPK pathway regulates cellular growth and proliferation. WNT and TGF-β pathways are both involved in mammary stem cell renewal. Down-regulation of TGF-β leads to a decline in MaSC number (Petersen and Polyak, 2010). A theme emerging from a variety of data is that stem cells exhibit characteristics of cells under stress (Covello et al., 2006; Mazumdar et al., 2010). An up-regulation of chaperones, ubiquitin/proteasome, DNA repair, and chromatin remodeling in LRECb are consistent with this characteristic and support our hypothesis that LRECb are enriched for MaSC.

Comparison of the transcript profiles of LRECe with those of ECe and LRECb supports classification of LRECe as progenitor cells. As with LRECb, presence of an HNF4A network and BrdU label retaining ability suggest that LRECe possess some stem cell attributes. However, up-regulation of metabolic enzymes and differentiation factors suggest that LRECe are more differentiated than LRECb. *XIST* is a non-coding RNA that inactivates one of the X-chromosomes in the early embryo and initiates gene repression and defines epigenetic transitions during development. Pluripotency genes (*NANOG*, *OCT4* and *SOX2*) cooperate to repress *XIST* (Navarro et al., 2008). Our mRNA data revealed low expression of *XIST* in LRECb and greater expression in LRECe and ECb, consistent with classification of LRECb as MaSC and LRECe as progenitor cells (Savarese et al., 2006). Finally, comparison of transcript abundance in LRECb vs. LRECe suggested up-regulation of the Notch pathway in LRECb, implying increased transduction of Notch signals in LRECb. The Notch pathway plays a critical role in cell fate determination of human mammary stem and progenitor cells (Dontu et al., 2004). In murine mammary gland, the Notch pathway constrains MaSC expansion and promotes proliferation and commitment to the luminal lineage (Bouras et al., 2008). Involvement of Notch signaling in putative MaSC (LRECb) along with pathways regulating stem cell expansion is consistent with the need to promote and balance the expansion of both MaSC and luminal epithelial cells during ductal mammogenesis.

Using laser microdissection and RNA-sequencing, Gordon and colleagues evaluated the transcriptomes of progenitor cells and differentiated cells in the gastrointestinal tracts of mice, discerned characteristics of these precursors and compared their molecular properties with those of stem/progenitor cells in other organs (Stappenbeck et al., 2003; Giannakis et al., 2006). The use of laser microdissection was efficacious and led to the identification of characteristics that are shared among various stem cells. Many of the molecular features of gastrointestinal and other adult stem cells that were identified are also evident in mammary LRECb (Table 4), supporting the hypothesis that the LRECb population is enriched for MaSC. Surprisingly, Gene Ontology-based analysis of transcripts that are differentially enriched in LREC and EC were inconsistent with the previously reported conclusion (Doherty et al., 2008) that stem cells exhibit increased expression of genes that are involved in nuclear function and RNA binding, while differentiated cells are enriched for expression of genes that are involved in extracellular space, signal transduction, and the plasma membrane (data not shown).

**Table 4:** **Attributes of murine somatic stem cells and bovine mammary LREC^1^**.

Murine somatic cells	LRECb	LRECe
**GEP, SiEP**		
Wnt/β-catenin signaling	+	
PI3K/AKT signaling		
TGF ß signaling	+	+
IGF-1 signaling	IGF2	
JAK/Stat regulation		
Cell Cycle (G1/S) Checkpoint	+	
NFκB signaling	+	+
**GEP, HSCs, NSCs, ESCs**		
Wnt/β-catenin signaling	+	
PI3K/AKT signaling		
Interferon signaling	+	
PDGF signaling		
T cell receptor signaling		
Estrogen receptor signaling	+	+
**GEPs, SiEPs, HSCs, NSCs, ESCs**		
Chemokine signaling	+	+
Integrin signaling		
SAPK/JNK signaling		
VEGF signaling		
IGF-1 signaling	IGF2	
B cell receptor signaling		
**SiEPs, HSCs, NSCs, ESCs**		
Wnt/β-catenin signaling		
IL4 and IL6 signaling	IL2, IL12	
Alanine and aspartate metabolism		
P38 MAPK signaling	+	
Integrin signaling		

*^1^Properties of murine somatic stem cells, assessed by Gordon and colleagues (Giannakis et al., 2006), and the relevance of these properties to bovine mammary LRECb. GEPs, gastric epithelial precursors; SiEPs, small intestine epithelial precursors; HSCs, hematopoietic stem cells; NSCs, neural stem cells; ESCs, embryonic stem cells*.

Our study provides supportive evidence that the stem cell niche lies in the basal layer of mammary epithelium. In this study, LREC and control cells were isolated from known locations within the mammary epithelium without previously destroying cellular microenvironments. However, LRECb were probably not in direct contact with the stroma, but were likely insulated by underlying cytoplasmic extensions from surrounding cells. The dissected LREC and control cells were adjacent or in close proximity to allow evaluations of potential cross-talk between putative MaSC and neighboring cells. Although potential signals were evident, additional research is necessary to elucidate such cross-talk. Furthermore, this analysis cannot account for signals that are derived from adjacent stromal cells, which were not interrogated. Microarray analyses of LRECb and LRECe identified features of LRECb that are reflective of MaSC residing in their stem cell niche. Distinct features of a stem cell niche, as discussed by Li and Xie (2005), are the presence of (1) cell adhesion molecules that provide anchorage for stem cells within the niche, (2) extrinsic factors within the niche that regulate stem cell behavior, and (3) factors that cause asymmetric cell division of the stem cell, that is upon cell division, one daughter cell is maintained in the niche as a stem cell (self renewal) and the other daughter cell leaves the niche to proliferate and differentiate. Recent studies also indicated that a stem cell niche elicits characteristics of hypoxic stress in stem cells, resulting in the induction of proteins of the family of hypoxia inducible transcription factors (*HIF*), such as *HIF1A* (up-regulated in LRECb), and targets *WNT*, *OCT4*, *IGF2* and Notch signaling molecules (Kaufman, 2010; Mazumdar et al., 2010). Mild hypoxia appeared to elicit expansion of mammary tumor stem cells via a mechanism mediated by *HIF1A* (Conley et al., 2012). In our study, we identified specific cell adhesion molecules, extrinsic growth factors and regulators, factors that promote asymmetric cell division, and hypoxia inducing factor as molecules that are prevalent in the stem cell niche of the basal epithelium (Table 1; Figure 6).

Finally, this research has identified molecular markers that are enriched in LREC. Transcripts encoding the nuclear proteins NR5A2, NUP153, FNDC3B, and HNF4A were identified as potential markers for MaSC, as were transcripts encoding surface proteins SAT2, THY1/CD90, CXCR4, SDPR, RTP3, CASR, GNB4, and DRD2. To our knowledge, none of these proteins have been tested or utilized as MaSC markers. Enrichment for MaSC has been based upon sorting for multiple markers, as no single marker has proved particularly efficacious. The utility of these markers for identification and for sorting of MaSC remains to be evaluated.

## Conclusion

Transcriptome analysis of LREC and mammary epithelial cell subpopulations has provided a framework for future studies of normal mammary epithelial cell development and homeostasis, and for the pathobiology of breast cancer. First, our data support the utility of long-term retention of DNA label as a means to identify an enriched population of progenitor cells. The data support the hypothesis that LRECs are enriched for MaSC and progenitor cells, with LRECb being enriched for progenitors with more stemness features (putative MaSC) and LRECe being enriched for more committed progenitors. Second, our data support the contention that the basal layer of the mammary epithelium provides for the MaSC niche. Lastly, we offer the first transcriptome profile of putative MaSC (LRECb) and progenitor cells (LRECe) excised from their *in situ* locations and we have identified potential novel biomarkers for these cells.

Insights into the biology of stem cells will be gained by further confirmation of candidate MaSC markers proposed by this study. Such confirmation requires an evaluation of the self renewal and differentiation potential of cells expressing these markers. Identification of appropriate biomarkers will provide a means to identify MaSC and will facilitate our understanding of MaSC functions in mammary development, homeostasis, and cancer. Specific cell surface markers will provide a means for future isolation of MaSC and investigations of their biology.

## Conflict of Interest Statement

The authors declare that the research was conducted in the absence of any commercial or financial relationships that could be construed as a potential conflict of interest.

## Supplementary Material

The Supplementary Material for this article can be found online at http://www.frontiersin.org/Cancer_Genetics/10.3389/fonc.2013.00021/abstract

Supplementary Table S1 **Microsoft excel worksheet listing transcripts whose abundance differs between cell types**. Comparisons are: LRECb vs. ECb, LRECe vs. ECe, LRECb vs. LRECe, and ECb vs. ECe.

Supplementary Table S2 **Microsoft Excel worksheet listing genes (partial list) enriched in LRECb vs. control cells (ECb) and their general functional categories**.

## Figures and Tables

**Figure A1:**
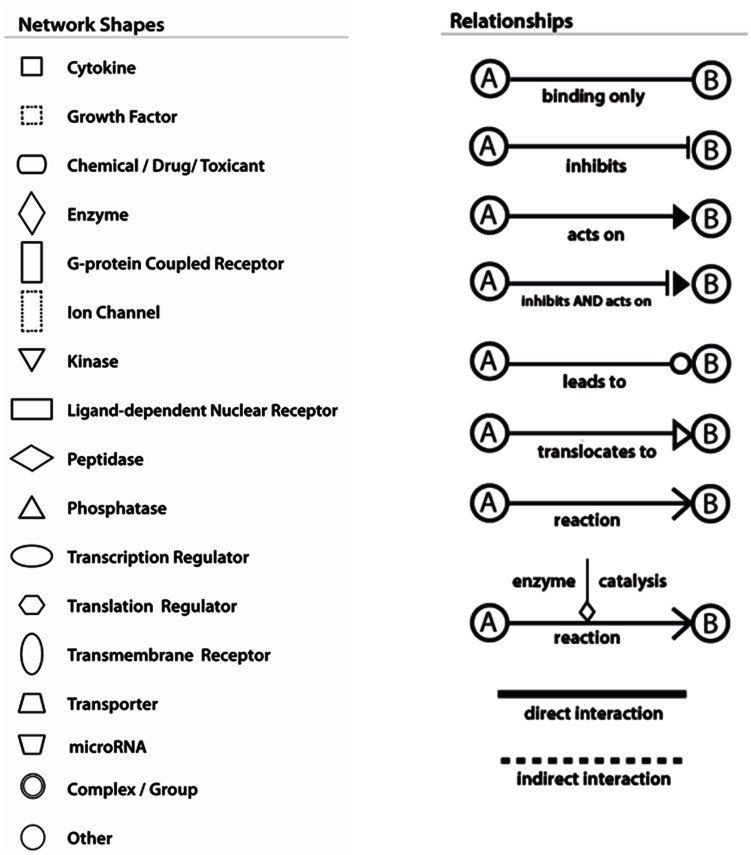
**JPG file defining the symbols and relationships depicted in IPA networks**.

## Footnotes

^1^http://uswest.ensembl.org/downloads.html

^2^www.ingenuity.com

^3^http://www.ncbi.nlm.nih.gov/geo/query/acc.cgi?acc=GSE31541.
